# Lipopolysaccharide derived from the digestive tract triggers an inflammatory response in the uterus of mid-lactating dairy cows during SARA

**DOI:** 10.1186/s12917-016-0907-1

**Published:** 2016-12-12

**Authors:** Muhammad Shahid Bilal, Juma Ahamed Abaker, Zain ul Aabdin, Tianle Xu, Hongyu Dai, Kai Zhang, Xinxin Liu, Xiangzhen Shen

**Affiliations:** College of Veterinary Medicine, Nanjing Agricultural University, Nanjing, 210095 People’s Republic of China

**Keywords:** Lipopolysaccharide, Sub-acute ruminal acidosis, Cow, LBP, Immune gene expression

## Abstract

**Background:**

The aims of the current study were to evaluate the inflammatory response in cow uterus and to explore the molecular mechanism triggered by high concentrate-induced subacute ruminal acidosis (SARA) in mid-lactating dairy cows. Twelve mid-lactating Holstein cows with an average weight of 455 kg were allocated into two groups subjected to two diets for 18-weeks either a low-concentrate (LC) group containing 4:6 (NDF: NFC) and a high-concentrate (HC) group containing 6:4 (non-forage carbohydrates, NFC): (neutral detergent fiber, NDF) ratio based on dry matter.

**Results:**

The HC group showed lower ruminal pH and higher lipopolysaccharide (LPS) concentrations in both the rumen and peripheral plasma compared to the LC group. The LPS concentrations in the rumen fluid and the peripheral plasma were found significantly increased in the HC group compared to the LC group. The concentrations of IL-1β, TNF-α and IL-6 were significantly higher in the HC group compared to the LC group. The uterus of SARA cows revealed elevated mRNA concentrations of nuclear transcription factors and pro-inflammatory cytokines, which confirmed the presence of inflammation. The occurrence of uterine inflammation was further validated by the increased protein expression of NF-κB-p65 and its active phosphorylated variant in the uterus of SARA cows. Similarly, the inflammatory genes TLR4, LBP, MyD88, TRAF-6, NF-κB, IL-6, IL-8, TNF-α and IL-1β were significantly upregulated in the uterus of the HC versus the LC group.

**Conclusion:**

Therefore, the results indicated that LPS derived from the rumen triggered the genes associated with inflammation in the uterus of mid-lactating dairy cows fed a high-concentrate diet, causing endometritis.

## Background

Feeding a high-concentrate diet for a longer period of time can cause subacute ruminal acidosis (SARA). It is a digestive disorder with a pH less than 5.6 lasted for more than 3 h per day [1, 2]. General clinical signs of SARA comprise of reduced dry matter intake (DMI), decreased ruminal pH and diarrhea [3, 4]. Another crucial outcome of SARA is the decline of milk quantity and quality [2, 3], and earlier studies also revealed that SARA decreased milk protein yield [4, 5]. The persistent consumption of a HC diet by dairy animals enhances the production of organic acids and significantly results in a decline in pH values of the rumen and intestine [5–7]. This decrease in pH due to excessive high concentrate diet [8] may affect the alterations of the type of fermentation [9] and the structure of the microbes in the rumen [10, 11]. Moreover, the decline in pH also perturbs the balance of microbial population in the rumen causing substantial release of free endotoxin (lipopolysaccharide, LPS) from gram-negative bacteria [12]. Due to possible alterations in permeability and disruption of the gastrointestinal barrier, LPS can be translocated into the blood stream [2, 13, 14]. There are also other reports revealed that elevated circulating LPS cause a systemic inflammatory response [15, 16]. Possibly, being an element of an immune response to LPS, SARA has been reported to increase the concentration of acute phase proteins in the peripheral blood, such as serum amyloid A (SAA) and haptogloblin (Hp) [2, 12, 14, 17]. The SAA has many functions but mainly alters innate immune responses, particularly in the migration of neutrophils and monocytes, while Hp has anti-inflammatory effects; LPS-binding protein (LBP) is activated by microbial infections and facilitates in the neutralization of LPS and in the triggering the release of pro-inflammatory cytokines, Such as TNF-α, IL-6 and IL-1β [6, 18–20]. The innate immune system is the first line of defence against invading pathogens; it involves various types of transmembrane and secreted molecules, recognized as pattern recognition receptors (PRR). Toll like receptors, such as TLR4, that join the LPS-LBP complex, are located on the outer surfaces of a variety of cells and contribute to the sensing of microbial pathogens in the intracellular endosomes and lysosomes [21, 22]. TLR4 recognizes LPS with the help of the LBP and cluster of differentiation antigen14 (CD14) [23, 24]. LPS activates myeloid differentiating factor 88 (MyD88) after joining with TLR4 on the host cell surface [25]. MyD88 activates TNF receptor-associated factor 6 (TRAF6), which stimulates the IκB kinase (IκK) complex, and phosphorylated NF-κB is consequently translocated from the cytoplasm to the nucleus [26, 27]. The NF-κB-dependent cascade pathway regulates the production of pro-inflammatory cytokines, including interleukin (IL)-1β, TNF-α, IL-6, chemokines (IL-8) and other inflammatory mediators [28]. The principal objectives of this study were to evaluate the inflammatory response in cow uterus and to explore the molecular mechanism triggered by high concentrate-induced SARA in mid-lactating dairy cows.

## Methods

### Animals, diets and experimental design

Twelve healthy multiparous Holstein cows, which were purchased from the Experimental Farm of Nanjing Agricultural University, with an average body weight of 455 kg were selected for the current study. The experimental cows were randomly divided into two groups, each group containing six cows: the high-concentrate (HC) group and the low-concentrate (LC) group. The HC group was provided a high-concentrate diet containing 6:4 (non-forage carbohydrates, NFC): (neutral detergent fiber, NDF) ratio, while LC group was fed low concentrate diet containing 4:6 (NDF: NFC) for the 18-week experimental period. The details of the nutritional ingredients and their compositions are shown in Table 1. The cows were surgically fixed with a ruminal fistula two weeks prior to the experiment. The cows were kept in individual tie stalls and feed was offered three times per day at 0400, 1200 and 2000 h. DMI was ~21.7 kg/d/head, and fresh drinking water was available to them during the entire experimental period. The experiment was started after the animals made a full recovery from the ruminal fistula fixation, and no cows showed any clinical signs of infection during the experiment.

**Table 1:** Ingredient and nutrient composition of diets

Ingredients, % of DM	LC^a^	HC^a^
Corn silage	30.0	20.0
Alfalfa	30.0	20.0
Maize	22.7	33.6
Wheat bran	5.1	15.0
Soybean meal	9.8	9.0
Calcium phosphate dibasic	0.9	0.5
Limestone	0.0	0.5
Salt	0.3	0.3
Premix^b^	1.0	1.0
Total	100.0	100.0
Nutritional Composition^c^		
NE MJ/kg	6.3	6.7
CP %	16.0	16.2
EE %	3.9	4.1
NDF %	37.7	31.9
ADF %	22.7	17.5
NFC %	33.4	40.3
Starch, %	25.3	32.2
Ca %	0.9	0.8
P %	0.4	0.4

^a^Treatment LC, 40% grain on DM basis; HC, 60% grain on DM basis

*NE* Net energy mega joules, *CP* crude protein, *EE* ether extract, *NDF* neutral Detergent fiber, *NFC* non-forage carbohydrates, *ADF* Acid detergent fiber

^b^The premix contained VA,1.900,000 IU/kg; VD, 250,000 IU/kg; VE, 3484.32 IU/Kg; Niacin, 4,000 mg/Kg; Cu, 1,200 mg/Kg; Fe, 525 mg/Kg; Zn, 13,000 mg/Kg; Mn, 5,500 mg/kg; I, 170 mg/Kg; Co, 50 mg/Kg; Se, 27 mg/Mg

^c^The calculated nutritional composition values

### Collection of samples and analysis

The rumen fluid was collected at one-hour intervals for 12 h daily for 3 continuous days during the 18^th^ week. Blood samples were collected 4 h after feeding on the sampling days (5, 6 and 7) of the 18^th^ week. 10 mL of rumen fluid was immediately centrifuged at 10,000 × g for 45 min, filtered through a disposable 0.22-μm filter and stored in Pyrogen free glass at −20 °C for LPS analysis. The blood samples were collected from the jugular vein into 5-mL vacuum tubes containing sodium heparin as an anticoagulant. The plasma was separated from the blood samples through centrifugation at 3000 × g at 4 °C for 15 min and stored at −20 °C to determine the concentrations of the pro-inflammatory cytokines IL-1β, IL-6, and TNFα in the peripheral blood. At the end of the experiment, the cows were slaughtered after overnight fasting. The slaughtering was performed according to the law of Jiangsu Provincial People’s Government, China and the study was approved by the Animal Ethics Committee (AEC) of Nanjing Agricultural University, China as described in the end of the manuscript. After slaughtering, the uterine tissues were removed from all the cows aseptically, as close to the cervix as possible for the analysis. All collected tissues were immediately frozen in liquid nitrogen and stored at −70 °C within 30 min of slaughtering [29].

### Determination of ruminal pH

Rumen fluids were taken through a rumen fistula at one-hour intervals up to 12 h on days 5, 6 and 7 of the 18^th^ week of the trial. The pH of rumen was measured each hour immediately following the sample collection by pH-meter (Sartorius, Basic pH Meter PB-10, PB-21, Goettingen, Germany).

### Determination of LPS

The concentration of LPS in the rumen fluid and plasma of the jugular vein was determined by Chromogenic Endpoint Limulus Amebocyte Lysate Assay Kits, CE64406 and CE80545 (Chinese Horseshoe Crab Reagent Manufactory Co., Ltd., Xiamen, China). Briefly, the pre-treated rumen fluid and plasma samples were diluted until their LPS concentrations were in the range of 0.1 to 1 endotoxin units (EU)/mL relative to the reference endotoxin and assayed as described by Gozho et al. [2, 6].

### Radioimmunoassay

Radioimmunoassay was used to calculate the concentrations of IL-6, IL-1β, and TNF-α in peripheral blood using radioimmunoassay (RIA) kits (IL-1β, C09DJB; IL-6, C12DJB; TNF-α, C06PJB; Beijing North Institute of Biological Technology, Beijing, China).

### RNA extraction and real time PCR (RT-q PCR)

Total RNA was prepared from 100 mg uterus tissue using TRIzol (Takara Co., Otsu, Japan) via homogenization on ice as described by the manufacturer’s instructions. The quality of mRNA was assessed by both agarose gel (1%) electrophoresis and spectrometry (A260/A280) using the Eppendorf Bio photometer Plus (Eppendorf AG, Hamburg, Germany). Only samples with a ratio between 1.8 and 2.1 were used in subsequent experiments. The first-strand cDNA was synthesized using 250 ng/μl of the total RNA template using Prime Script RT Master Mix Perfect Real Time (Takara Co., Otsu, Japan) according to the manufacturer’s instructions.

Quantitative real-time PCR (qRT-PCR) amplification was performed to evaluate the expression of the selected genes. The primers for genes IL-1β, TLR4, IL-6, IL-8 [30], and LBP [31] were used according to previously published work, while MyD88, TNF-α, TRAF6, and NF-κB were designed using the Premier primer 5.0 software. The primers were assessed for their amplification efficiencies. The reaction conditions included 2 μl of cDNA and 0.4 μM primers in a total volume of 20 μl of super mix. Thermal cycling parameters consisted of initial denaturation at 95 °C for 15 s, followed by 40 cycles of annealing at 95 °C for 5 s and primer extension at 60 °C for 31 s. All the reactions were run in triplicate. Each cDNA sample was amplified using SYBR Green (Takara Co., Otsu, Japan) in the ABI 7300 Fast Real-Time PCR System (Applied Bio systems, USA).

The data were normalized to the mean of housekeeping gene β-actin to control the unpredictability in expression levels and were analysed using *R* = 2^-ΔΔCt^ method (Ct _target gene_ - Ct _*β* -actin_) treatment - (Ct _target gene_ - Ct _*β* -actin_) control), as previously depicted [32]. The primer sequences used in this study are presented in Table 2.

**Table 2:** The list of primer sequences used for amplification of qRT-PCR

Gene	Forward primers	Reverse primers	Accession number	Product size (bp)
TLR-4	GGACCCTTGCGTACAGGTTG	GGAAGCTGGAGAAGTTATGGC	NM_174198.6	244
TRAF-6	GCGGCCTTCAAGTTAGGAGA	TCATCAACTGCTCGTTCGGG	NM_001034661.2	141
NF-*κ*B	ACGATCGTCACCGGATTGAG	GGTGCTGAGAGATGGCGTAA	XM_005699996.1	194
β-actin	CTCTTCCAGCCTTCCTTCCT	GGGCAGTGATCTCTTTCTGC	AY141970	178
IL-6	GGAGGAAAAGGACGGATGCT	GGTCAGTGTTTGTGGCTGGA	EU276071.1	226
IL-8	CCTCTTGTTCAATATGACTTCCA	GGCCCACTCTCAATAACTCTC	NM_173925.2	170
TNF-α	GCTCTTACCGGAACACTTCG	GGACACCTTGACCTCCTGAA	NM_173966	238
IL-1β	AACCGAGAAGTGGTGTTCTGC	TTGGGGTAGACTTTGGGGTCT	NM_174093	167
MyD88	AAAGCCCGAGTGTTTTGATG	TCACATTCCTTGCTTTGCAG	NM_001014382	234
LBP	GCAAGATCACTGGATTCTTGGA	AAAACAGGAAGTCCTTGTGGATC	NM_001038674.2	228

### Western blotting analysis

The protein was extracted from 100 mg uterus tissue with radio immunoprecipitation assay (RIPA) buffer using a Dounce Homogenizer (Polytron PT 1200 E, Switzerland). After 30 min of incubation on ice, cell extracts were subjected to centrifugation (12,000 × g) at 4 °C for 15 min to obtain cell protein. The supernatant was assayed for protein concentration using the bicinchoninic acid (BCA) protein assay kit (Pierce, Rockford, IL, USA) and was adjusted to 4 μg/μl. Equal amounts of proteins were isolated using 10% SDS-polyacrylamide gel electrophoresis (PAGE) and shifted to nitrocellulose membrane (NC) (Bio sharp, China) at 4 °C. Then, 10% non-fat dry milk was used to block the nitrocellulose membrane. The membrane was incubated at 4 °C overnight with monoclonal goat antibodies against NF-κB and GAPDH (Santa Cruz Biotechnology, Santa Cruz, CA, USA, diluted by 1:200), (Bio world, CA, USA, diluted by 1:10000), respectively. After incubation, the membrane was washed and incubated with horseradish peroxidase-conjugated goat anti-rabbit secondary antibodies. Immunoreactive proteins were detected by chemiluminescence using an ECL reagent (Super Signal West Pico Trial Kit, Pierce, USA) and subsequently autoradiographed. The results were quantified using Bio-Rad Gel Doc 2000™ Systems (Bio-Rad, USA) and analysed with Bio-Rad TDS Quantity One software (Bio-Rad). The relative quantities of proteins were evaluated by a densitometer and expressed as absorbance units (AU).

### Statistical analyses

The mixed procedure of SAS was used to analyze pH with a repeated measures design performed with SAS software (SAS version 9.2, SAS Institute Inc.). The effects of cows were considered random and the effects of diet and time were considered as fixed factors. The time within treatments and cows were considered as repeated measurements and the residuals for each variable were used to assess the normality. The days were averaged within a cow before the analysis for all the data including the pH, radioimmunoassay, ruminal LPS, plasma LPS and the gene expression data were assessed by independent *t*-test and expressed as mean ± SEM. Differences were concluded to be significant at *p* < 0.05.

## Results

### Rumen pH and LPS content of rumen fluid and blood plasma

The results of ruminal pH fed a high concentrate diet were published previously by Xu et al. [33] and he confirmed the presence of SARA by measuring the ruminal pH at different time intervals. In this study, consuming a high-concentrate diet (HC group) for 18 weeks resulted in a significant decline in ruminal pH (<5.6) than that in the LC group (*p* < 0.05) and lasted for more than three hours [2]. The effect of diet × hours on pH was significant (0.05); however no effect was observed on hours alone.

The LPS concentrations in the rumen fluid and the jugular vein plasma were significantly higher in the cows fed a high-concentrate diet compared to cows fed a low-concentrate diet. The concentration of LPS in the rumen fluid of the HC group was 79040 EU/mL, compared to 47170 EU/mL in the LC group (*p* < 0.01); similarly, in the peripheral plasma, the LPS level in the HC group was 860 EU/mL, whereas it was 470 EU/mL in LC group (*p* < 0.001), which indicated that the LPS level was significantly increased in the HC group relative to the LC group (Table 3).

**Table 3:** LPS concentration in rumen and plasma of dairy cows fed low concentrate (LC) and high concentrate (HC)

LPS concentration (EU/mL)	Treatment^a^			
	LC	HC	SEM^b^	*p*-Value
Rumen LPS	47170	79040	7966.25	<0.01
Jugular vein Plasma LPS	470	860	81.26	<0.001

^a^ *HC* high concentrate diet, *LC* low concentrate diet, *EU* endotoxin unit

^b^ *SEM* Standard error of the mean between the two groups

The LPS data were compared using Student’s *t*-test between HC and LC groups

*P* ≤ 0.05 was considered significant

### Levels of pro-inflammatory cytokines in the peripheral blood

**Fig. 1:**
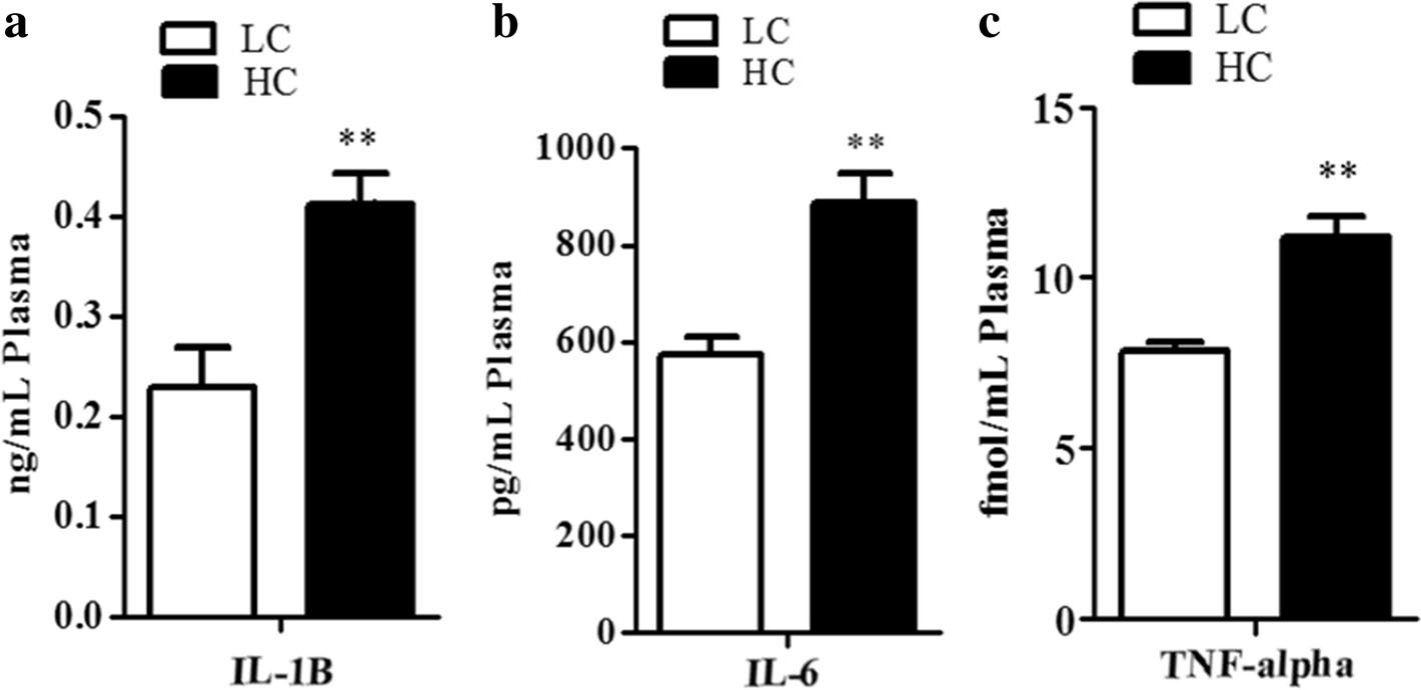
**a**-**c** The concentration levels of TNF-α, IL-1β, and IL-6 in the peripheral blood. The data were expressed as the mean ± SEM.; asterisks indicate the differences between the low-concentrate (LC) group and high-concentrate (HC) group. (*p < 0.05, **p < 0.01, *n* = 12)

The results of the measurement of pro-inflammatory cytokines, together with IL-1β, IL-6 and TNF-α are shown in Fig. 1. The concentration of IL-1β was significantly higher in the HC group compared to the LC group (*p* < 0.007) (Fig. 1a), the concentration of IL-6 was significantly increased in the HC group versus LC group (*p* < 0.004) (Fig. 1b) and the concentration of TNF-α was elevated in the HC group relative to the LC group (*p* < 0.002) (Fig. 1c). Generally, the concentration of pro-inflammatory cytokines in the peripheral blood was significantly higher in the HC group compared to the LC group.

### Expressions of genes involved in the inflammation of the uterus

**Fig. 2:**
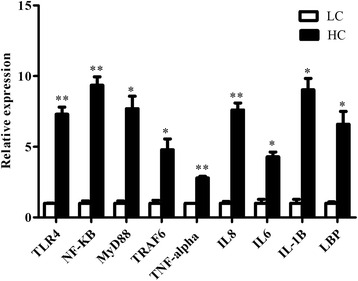
The uterine gene expression profile analysed by qRT-PCR. The genes involved in transcriptional regulation were measured in the uterine tissues. The error bars indicate the standard error of the mean and ** indicates significance at *p* < 0.01

SARA significantly increased the expression level of several genes involved in the inflammation of the uterus as shown in Fig. 2. The gene expression of TLR4 (*p* < 0.006) and MyD88 (*p* < 0.01) in the HC group was significantly up-regulated compared to the LC group. Similarly, TRAF-6 (*p* < 0.04) and NF-κB (*p* < 0.005) were highly expressed in the HC group relative to the LC group. The gene expression of TNF-α (*p* < 0.005) and IL-8 (*p* < 0.005) was significantly increased. The gene expression of IL-1β (*p* < 0.01), IL-6 (*p* < 0.01) and LBP (*p* < 0.03) was also significantly higher in the HC group compared with the LC group.

### Expression of NF-κB protein in the cow uterus of the HC group and LC group

**Fig. 3:**
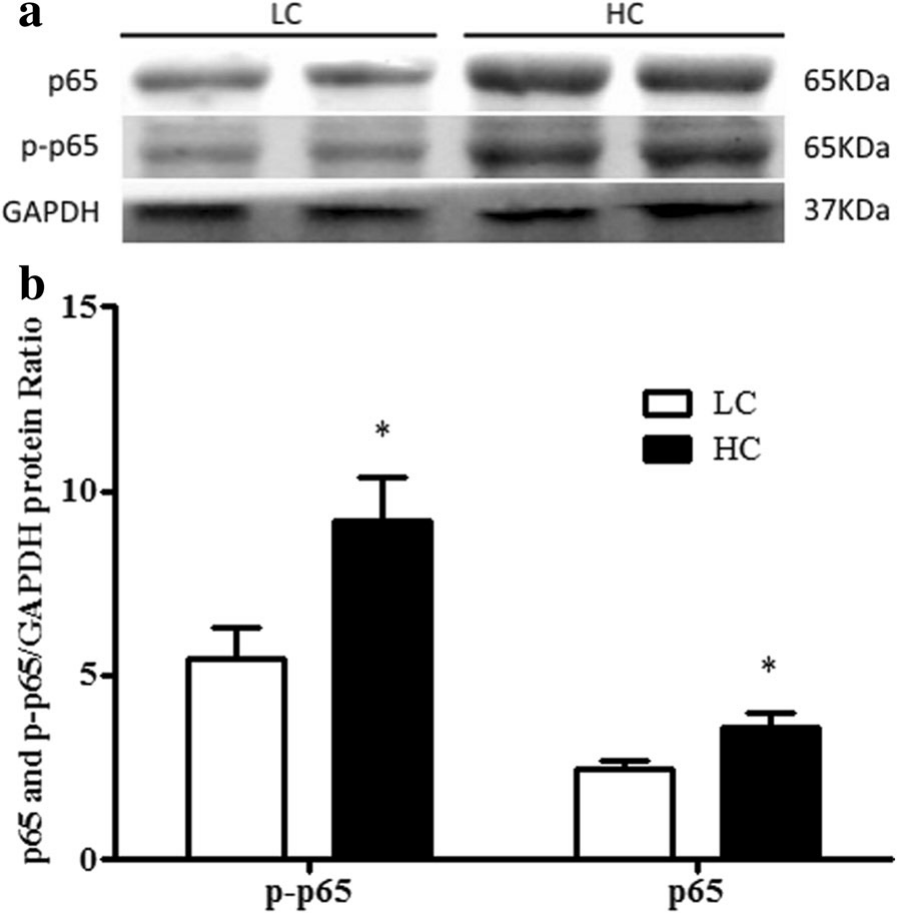
**a** The western blotting assay of NF-κB (p65) and phosphorylated-p65 protein in the uterus. The NF-κB contents in uterus were evaluated through western blot of the low-concentrate (LC) group and high-concentrate (HC) group. **b** The quantities of proteins are measured as arbitrary units relative to GAPDH; fold alterations in NF-κB (p65) and phosphorylated NF-κB (p-p65). The data were expressed as the mean ± SEM., asterisks indicate the differences between the high-concentrate (HC) group compared to the low-concentrate (LC) group (** *p* < 0.01)

Western blot analysis demonstrated that high concentrate diet-induced inflammation through the increaseduterine concentrations of the NF-κB (p65) proteins in the HC group compared with the LC group (*p* < 0.03; Fig. 3a). Importantly, the amount of phosphorylated NF-κB (p-p65) was higher in the uterus of the HC group than in the LC group (*p* < 0.04; Fig. 3b). Therefore, it was confirmed that NF-κB was activated in HC group in the TLR4 signalling pathway.

## Discussion

The present study assessed the uterine inflammation at the molecular level in dairy cows. The inflammatory response in the uterus due to circulating LPS derived from rumen triggered the expression of pro-inflammatory cytokines through TLR4 signaling. To evaluate the inflammatory response in cow uterus and to explore the molecular mechanism triggered by high concentrate diet, SARA was confirmed by measuring the pH of the rumen. Xu et al. [33] also confirmed the presence of SARA by measuring the ruminal pH at different time intervals. In our study, the low rumen pH (>5.6) lasted for 223 min and high concentrations of LPS were observed in the rumen fluid and peripheral blood. The recent reports also indicated the successful induction of SARA, if theduration of low rumen pH was more than 180 min/day [22, 31]. However, there is little literature revealing that SARA not only depends on pH, but also on the alteration in the microbial population that develops secondarily to the feed type [34]. As a matter of fact, low ruminal pH values led to the lysis of gram-negative bacteria which released LPS into the blood circulation [31, 35]. Consistent with previous studies in dairy cows and feedlot, our results presented higher concentrations of LPS in the peripheral plasma (860 EU/mL) and ruminal fluid (79040 EU/mL) of cows fed a high concentrate die trelative to the peripheral plasma of the LC group [14, 36]. Moreover, we also found that high concentrate diet feeding increased the plasma concentrations of pro-inflammatory cytokines (IL-1β, TNF-α and IL-6). Similar results have been reported by other researchers [6, 19, 37]. The increment in pro-inflammatory cytokines concentration in the plasma may provide confirmation for LPS translocation into the blood circulation and eliciting of inflammatory responses. This implies that inflammation in the HC group was due to the high level of LPS in the bloodstream, which in turn triggers the pro-inflammatory gene to cope with stress conditions [37].

The RT-qPCR-based findings revealed the presence of inflammation-associated markers in the uterus of SARA-induced cows and confirmed that the dairy cows suffered from uterine inflammation at the molecular level. According to our results, the increased expression of TLR4 indicated that TLR4 recognized the LPS and triggered the inflammatory pathway [9, 38]. Identification of inflammatory-stimuli by the innate immune system is organized by PPR that identify external stimuli for example pathogen associated molecular patterns (PAMPs) [39]. Furthermore, our research demonstrated the increased expression of LBP, MyD88, and TRAF6 in the uterus; which provides more clues that the TLR4 signaling pathway was stimulated by LPS [25, 40]. Previous studies demonstrated that as a main transcription factor, NF-κB may bind to the promoter regions of the many inflammatory genes to regulate their transcription activities [37, 41]. Similarly, in the current study, NF-κB expression was increased in the HC group, which also confirmed the activation of TLR4 signalling pathway. Our results of increased expression of pro-inflammatory cytokines (IL-1β, TNF-α and IL-6) and chemokine (IL-8) in the HC group due to SARA-induced LPS were consistent with the previous studies [25, 42–46].

The western blot results further confirmed that the expression levels of NF-κB (p65) and phosphorylated p65 proteins were distinctly increased in the HC group compared to the LC group. Taken together, the results suggested that LPS triggered the activation of TLR4 signalling, which resulted in an inflammatory response in the uterus of cows suffering from SARA. Conclusively, LPS activates TLR4-NF-κB signalling. Together with the existing study and the previous reports [33, 37], the protein expressions of NF-κB (p65) and p-p65 were magnified in the HC group.

## Conclusions

According to our results, long-term feeding of high concentrate diet causes uterine inflammation in mid-lactating dairy cows. The inflammatory response in the uterus induced by increased level of circulating LPS triggers the expression of pro-inflammatory cytokines in the uterus through the TLR4-NF-κB signalling pathway.

## Abbreviations

AMP: Antimicrobial peptides
APP: Acute phase proteins
CD14: Cluster of differentiation antigen14
EECs: Endometrial epithelial cells
GIT: Gastrointestinal tract
HC: High concentrate
IL-1 α: −1 β, −6, Interleukin-1 α, 1 β, −6
IκK: IκB kinase
LBP: LPS-binding protein
LC: Low concentrate
LPS: Lipopolysaccharide
MyD88: Myeloid differentiating factor 88
NF-κB: Nuclear factor kappa-light-chain-enhancer of activated B cells
PAGE: Polyacrylamide gel electrophoresis
PAMPs: pathogen-associated molecular patterns
PRR: Pattern recognition receptors
qRT-PCR: Quantitative real-time PCR
SARA: Subacute ruminal acidosis
TLRs: Toll-like receptors
TRAF6: TNF receptor-associated factor 6
